# Polyacrylonitrile-Nanofiber-Based Gel Polymer Electrolyte for Novel Aqueous Sodium-Ion Battery Based on a Na_4_Mn_9_O_18_ Cathode and Zn Metal Anode

**DOI:** 10.3390/polym10080853

**Published:** 2018-08-02

**Authors:** Yongguang Zhang, Zhumabay Bakenov, Taizhe Tan, Jin Huang

**Affiliations:** 1 School of Materials and Energy, Synergy Innovation Institute of GDUT, Guangdong University of Technology, Guangzhou 510006, China; ygzhang126@126.com; 2 Institute of Batteries LLC, National Laboratory Astana, School of Engineering, Nazarbayev University, 53 Kabanbay Batyr Avenue, Astana 010000, Kazakhstan; zbakenov@nu.edu.kz

**Keywords:** aqueous sodium-ion battery, cathode, gel polymer electrolyte, Na_4_Mn_9_O_18_ nanorod, polyacrylonitrile nanofiber

## Abstract

A gel polymer electrolyte was formed by trapping an optimized Na^+^/Zn^2+^ mixed-ion aqueous electrolyte in a polyacrylonitrile nanofiber polymer matrix. This electrolyte was used in a novel aqueous sodium-ion battery (ASIB) system, which was assembled by using a zinc anode and Na_4_Mn_9_O_18_ cathode. The nanorod-like Na_4_Mn_9_O_18_ was synthesized by a hydrothermal soft chemical reaction. The structural and morphological measurement confirmed that the highly crystalline Na_4_Mn_9_O_18_ nanorods are uniformly distributed. Electrochemical tests of Na_4_Mn_9_O_18_//Zn gel polymer battery demonstrated its high cycle stability along with a good rate of performance. The battery delivers an initial discharge capacity of 96 mAh g^−1^, and 64 mAh g^−1^ after 200 cycles at a high cycling rate of 1 C. Our results demonstrate that the Na_4_Mn_9_O_18_//Zn gel polymer battery is a promising and safe high-performance battery.

## 1. Introduction

The lithium-ion battery (LIB) is the most preferred technology for application in portable electronic devices [1,2]. However, facing the ever-increasing demand for energy storage devices, there is a huge stress on the lithium supply chain, resulting in a rather unsustainable increase in the raw material cost [3,4,5]. Additionally, the use of liquid organic electrolytes in the existing LIB systems usually brings forward safety concerns due to their high flammability and toxicity [6]. Polymer electrolytes with high ionic conductivity and energy density can overcome the disadvantages of the liquid electrolyte, therefore, they have received much attention due to their potential applications in electrochemical devices [7,8]. In 1975, Feuillade and Perche developed a polymer electrolyte by adding polyacrylonitrile (PAN) and polyvinylidene fluoride (PVDF) to increase the ionic conductivity at room temperature [9]. The PAN-based electrolytes have advantages over other polymer electrolytes due to their good mechanical properties and high ionic conductivity [10]. Therefore, gel polymer electrolytes have attracted considerable attention as an alternative to the liquid systems [11]. Along with the safety enhancement, gel polymer membranes serve both as conducting media and as separators, a combination which provides advantages of much simplified fabrication and modularity in design of batteries [12]. J. Prakash et al. found that the battery thermal runaway in LIBs occurs with increase of temperature during cycling corresponding to the SEI film breakdown and thermal decomposition of its components [13]. J. R. Dahn and E. W. Fuller showed by thermal gravimetric analysis that the positive electrodes (LiCoO_2_, LiNiO_2_ or LiMn_2_O_4_) of commercial LIBS are metastable and liberate oxygen when they are heated in air or in inert gas rising remarkable safety risks [14]. J. Prakash et al. expound contributions of reversible and irreversible heats to the overall heat generated during charge and discharge cycling by a continuum model [15]. To solve the thermal runaway problem, C. W. Lee et al. proposed the electrolyte containing a flame-retardant additive hexamethoxycyclotriphosphazene [NP(OCH_3_)_2_]_3_ to improve electrochemical performance of the cell [16]. Such approaches can also improve thermal stability and hence nonflammability of the electrolyte. However, the use of such additives and other techniques, for example, the use of ceramic separators (which are usually fragile and reduce the system’s conductivity), the flammability of the organic electrolytes cannot be completely avoided. Aqueous electrolyte could eliminate flammability issue, although there are some disadvantages of these electrolytes such as the lower operating potential which needs to be solved for enhancing the power density and resolve the tradeoff between safety and battery performance. These considerations lead to an exciting way to build a novel aqueous sodium-ion gel polymer electrolyte battery system (ASGB), minimizing flammable liquid leakage, while maintaining high ionic conductivity.

In this work, we report on preparation of a polyacrylonitrile (PAN)-based gel polymer electrolyte via electrospinning at high voltage, which transformed the polymer solution into uniform and slender nanofibers [17]. A previous study in this field had reported a novel aqueous lithium-ion//zinc (Zn/LiMn_2_O_4_) rechargeable battery built without relying on the typical rocking-chair mechanism of LIB’s cathode and anode. The cathode reaction involves intercalation/de-intercalation of lithium-ion into/from the LiMn_2_O_4_, while the anode reaction consists of dissolution/deposition of Zn^2+^ on the metallic Zn foil. This aqueous system exhibits an excellent cyclability and rate capability [18,19]. Inspired by these results, we herein built a novel Zn/Na_4_Mn_9_O_18_ battery with an optimized Na^+^/Zn^2+^ mixed-ion electrolytes. In this battery system, a nanorod-like Na_4_Mn_9_O_18_ prepared by a hydrothermal soft chemical reaction is used as a cathode, demonstrating a long cycle span as well as a good rate capability.

## 2. Materials and Methods

### 2.1. Materials Preparation

Na_4_Mn_9_O_18_ was prepared by the hydrothermal soft chemical reaction (HSCR) with a high NaOH concentration. At first, 25 mL of aqueous solution containing 3.0 mol L^−1^ NaOH and 0.1 mol L^−1^ KMnO_4_ was prepared. Then, an equal volume of 0.28 mol L^−1^ MnSO_4_ aqueous solution was added to the previous solution with continuous stirring which quickly resulted in the formation of a dark brown precipitate. The precipitate was separated by filtration and washed by deionized water, and then dried in air at room temperature for 24 h to obtain the Na-birnessite precursors. To synthesize Na_4_Mn_9_O_18_, 3.0 g wet Na-birnessite precursor was added into 75 mL of a high concentration (15 mol L^−1^) NaOH solution and stirred for 20 min to obtain a brown suspension. Further, the suspension was poured into a 100 mL stainless steel autoclave with a Teflon liner (Zhongkaiya, Jiangsu, China), and heated at 180 °C for 18 h. Finally, the product was washed repeatedly with deionized water to eliminate the excessive NaOH followed by drying at 60 °C.

The PAN-nanofiber membranes were prepared by the electrospinning method. PAN was vacuum dried at 60 °C for 24 h prior to use. Firstly, a certain amount of PAN (Sigma-Aldrich, St. Louis, MO, USA, *M_W_* = 150,000 g mol^−1^) was dissolved in the *N*,*N*-Dimethylformamide (DMF, ≥99%, Aladdin, Shanghai, China) to form a homogeneous 12.5 wt. % solution under stirring for 24 h at room temperature. Then the polymer solution was electrospun by loading into a 10 mL plastic syringe, having a flow rate of 4 mL h^−1^ under a high voltage of 20 kV at room temperature. The distance between the syringe tip (having a diameter of 0.51 mm) and the collector plate of aluminum was 21 cm. The electrospun membranes were finally obtained on the aluminum foils after drying in a vacuum oven at 80 °C for 12 h. Finally, the prepared PAN-nanofiber membranes were activated by immersing in an aqueous sodium electrolyte solution consisting of 1 M Na_2_SO_4_ and 0.5 M ZnSO_4_ in water with pH = 4 at room temperature for 12 h. The PAN-nanofiber-based gel polymer electrolyte was obtained after wiping the surface of the membranes from the excess of the liquid electrolyte.

### 2.2. Physical Characterization

The crystalline structure of Na_4_Mn_9_O_18_ nanorods was analyzed using X-ray diffraction (XRD, D8 Discover, Bruker, Karlsruhe, Germany, Cu-Ka, λ = 0.154 nm). Brunauer-Emmett-Teller (BET, V-Sorb 2800P, Gold APP Instrument Corporation China, Beijing, China) tests were performed to analyze the specific surface area and porosities of the samples. The morphologies and structure of the samples were observed using scanning electron microscopy (SEM, Hitachi S-4800, Hitachi Limited, Tokyo, Japan) and high-resolution transmission electron microscopy (HR-TEM, JEOL2100, JEOL, Tokyo, Japan), respectively. Surface elemental analysis was done by the energy dispersive spectroscopy (EDS) module of the HR-TEM apparatus. Thermal runaway of the cells was investigated by means of differential scanning calorimetry (DSC Q20, TA Instruments, New Castle, DE, USA) with an open pan system under nitrogen purge at 30–40 mL min^−1^. Approximately 6 mg of PAN-based gel polymer electrolyte was placed inside the DSC pan for thermal analysis. The non-isothermal studies were performed at a scan heating rate of 10 °C min^−1^. Tensile strain of the samples was measured by dynamic mechanical analysis (DMA, Q800, TA Instruments, New Castle, DE, USA).

### 2.3. Electrochemical Characterization

The cathodes were made by casting the slurry of Na_4_Mn_9_O_18_, polyvinylidene fluoride and acetylene black in *N*-methyl-2-pyrrolidinone on carbon foil following a weight ratio of 8:1:1, and air dried at 75 °C for 12 h. The carbon foil and zinc foil were cut into disc electrodes (15 mm) and used as the cathode and anode, respectively. The CR2025 coin-type batteries were assembled by placing the PAN-nanofiber-based gel polymer electrolyte between the anodes and cathodes. The galvanostatic charge and discharge tests were carried out using a multichannel battery tester (BTS-5V5mA, Neware, Shenzhen, China) between 1.0 and 1.85 V (vs. Zn/Zn^2+^). The VersaSTAT electrochemical workstation (Princeton, VersaSTAT 4, Ametek, PA, USA) was used for cyclic voltammetry (CV) measurements of the cells. CV was conducted at different scan rates ranging from 1 to 2 V (vs. Zn/Zn^2+^).

## 3. Results and Discussion

**Figure 1:**
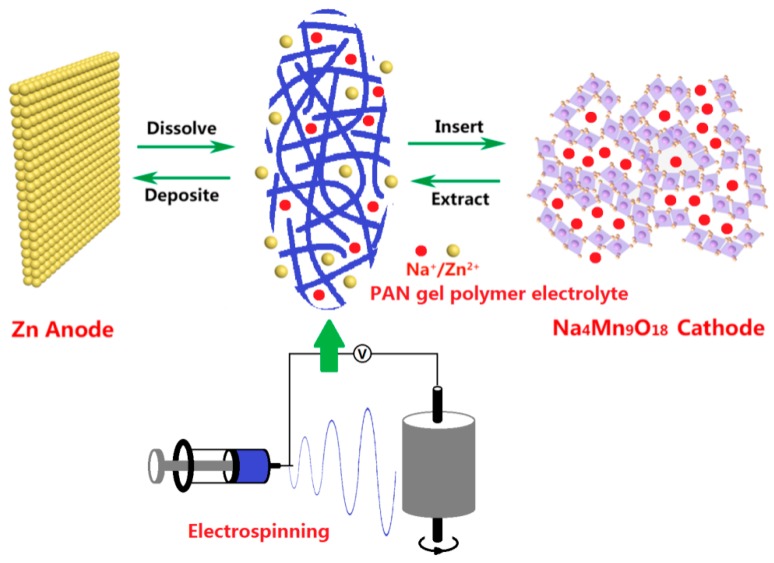
Schematics of mechanism of Zn/Na_4_Mn_9_O_18_ aqueous battery.

The electrochemical mechanism of Zn/Na_4_Mn_9_O_18_ battery operation is illustrated in Figure 1. When battery is charged, sodium ions are removed from Na_4_Mn_9_O_18_ cathode and dissolved in the electrolyte accompanied by liberation of electrons. This is accompanied by the deposition of zinc from the electrolyte on the surface of zinc anode. During the discharge process, Zn is oxidized and dissolved into the electrolyte, while sodium ions are inserted into the cathode to reversibly form Na_4_Mn_9_O_18_ [3]. The electrochemical reactions in the battery are schematically can presented by the following Equations (1)–(3):

The reaction at cathode:
  Na_4_Mn_9_O_18_ ⇔ Na_4−x_Mn_9_O_18_ + xNa^+^ + xe^−^ (1)
The reaction at anode:
  Zn^2+^ + 2e^−^ ⇔ Zn (2)
The total reaction:
  2Na_4_Mn_9_O_18_ + xZn^2+^ ⇔ 2Na_4−x_Mn_9_O_18_ + 2xNa^+^ + xZn (3)

**Figure 2:**
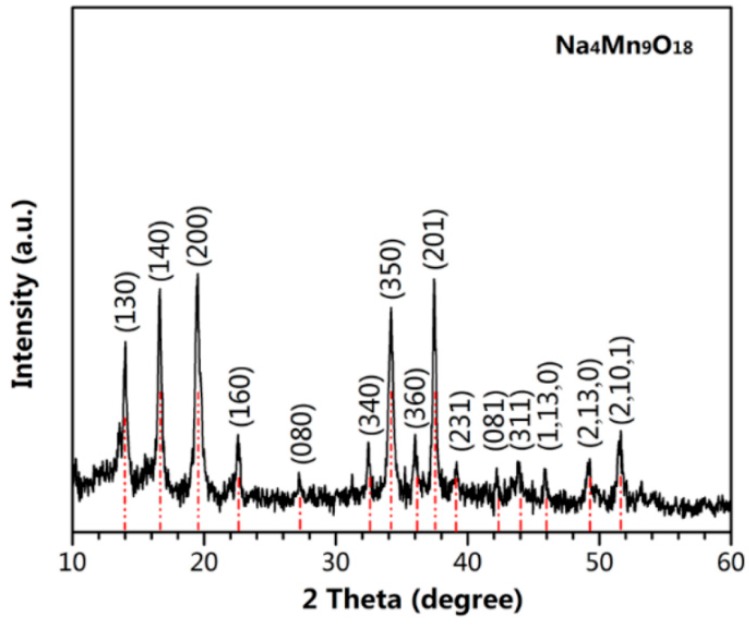
XRD pattern of Na_4_Mn_9_O_18_.

The XRD patterns of Na_4_Mn_9_O_18_ synthesized by HSCR method are displayed in Figure 2. The diffraction peaks are in good agreement with the JCPDS (The Joint Committee on Powder Diffraction Standards) Card number 27-0750 [20]. No impurity peaks were observed. These results agree well with the previous reports [21].

**Figure 3:**
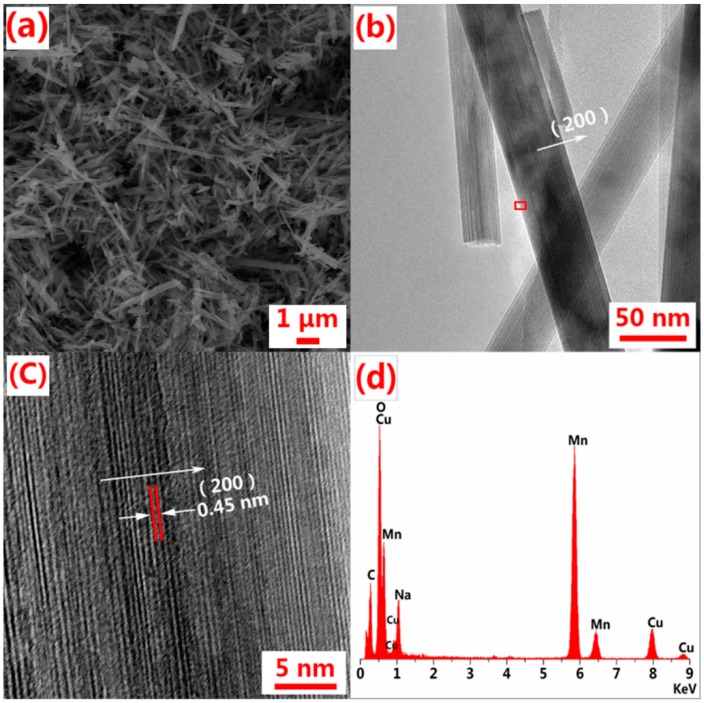
(**a**) SEM and (**b**) TEM image of Na_4_Mn_9_O_18_; (**c**) HR-TEM image and (**d**) EDS spectrum of Na_4_Mn_9_O_18_.

The morphology and structure of the prepared Na_4_Mn_9_O_18_ were confirmed by SEM and TEM, as displayed in Figure 3. Figure 3a shows that the Na_4_Mn_9_O_18_ grew anisotropically into nanorod crystal structure. The TEM image of Na_4_Mn_9_O_18_ in Figure 3b shows a rod-shaped structure with a smooth surface. The HR-TEM image in Figure 3c acquired from the red box in Figure 3b shows the vertical lattice fringe to be 0.45 nm, which corresponds to the (200) crystallographic plane of Na_4_Mn_9_O_18_. Interestingly, EDS spectrum of the Na_4_Mn_9_O_18_ nanorods (shown in Figure 3d), suggests a Na/Mn molar ratio of ~0.44.

**Figure 4:**
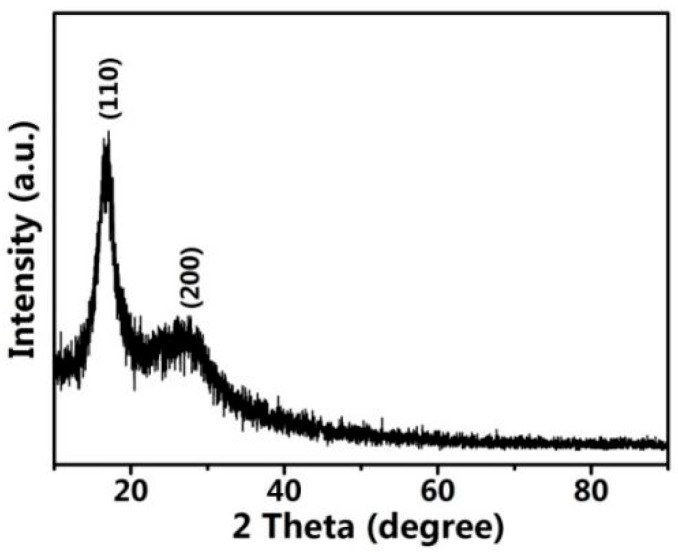
XRD pattern of the PAN-nanofiber membranes.

The XRD patterns of the PAN-nanofiber membranes are shown in Figure 4. A strong diffraction peak around 17° belonging to the (110) crystalline plane of a hexagonal structure could be clearly seen. Furthermore, the sample displays another weak diffraction peak around 29°, which is related to the (200) plane. The two obvious diffraction peaks suggest a semi-crystalline structure of the PAN-nanofiber membranes due to mixing of crystalline and amorphous phases [22,23].

**Figure 5:**
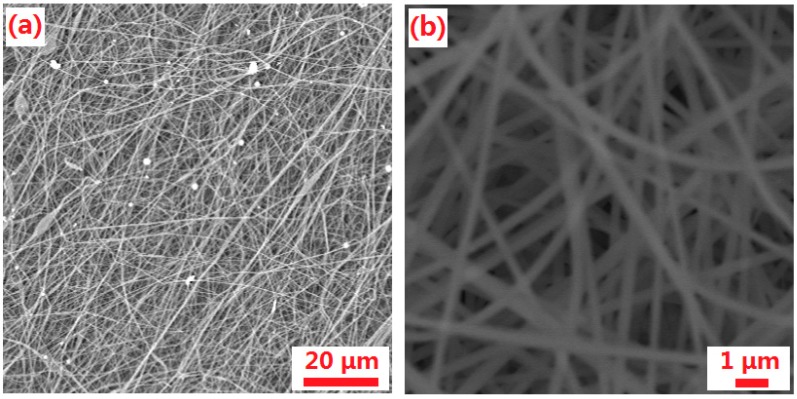
(**a**,**b**) SEM images of the PAN-nanofiber with different magnification.

The SEM images of the PAN-nanofiber membranes prepared by electrospinning are shown in Figure 5a. The electrospun membranes are composed of a three-dimensional crosslinking network structure with extremely regular nanofibers. The nanofibers are smooth and uniform, which positively affects the mechanical properties (strength) of the membrane [24,25].

**Figure 6:**
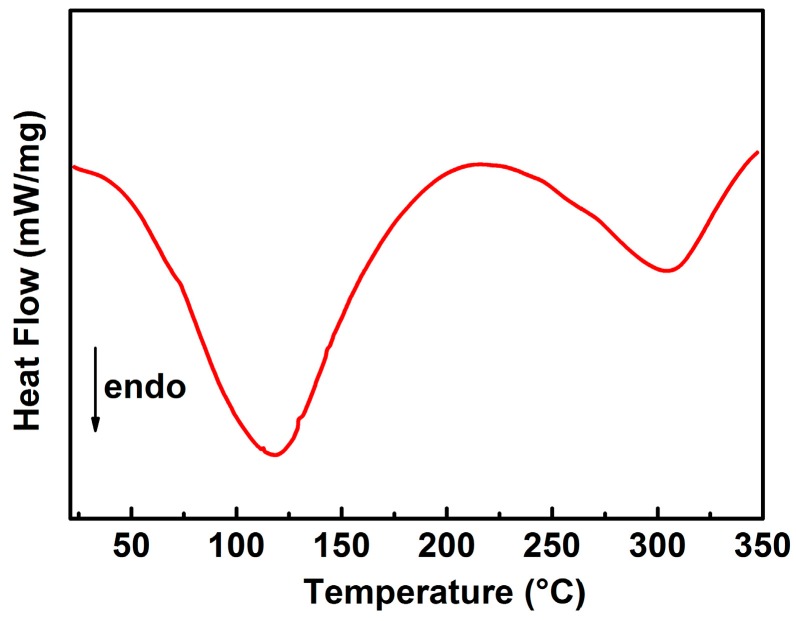
DSC curve of PAN-based gel polymer electrolyte.

Figure 6 shows the DSC thermogram of PAN-based gel polymer electrolyte (before immersed in sodium electrolyte solution) in the temperature range of 40–350 °C. From DSC, one endothermic peak could be observed around 117 °C (*T_g_*) corresponding to glass transition temperature and another endothermic peak could be observed around 305 °C (*T_m_*) corresponding to the melting temperature of the PAN-nanofiber membranes. When the prepared PAN-nanofiber membranes were immersed in an aqueous sodium electrolyte solution, the liquid uptake (%) was determined using the relation (*W*_2_ − *W*_1_) × 100/*W*_1_, where *W*_1_ and *W*_2_ denote the weights of PAN before and after absorbing the sodium solution, respectively. The result shows that the PAN-nanofiber membrane exhibited a high ability to absorb liquid electrolyte exceeding 65 wt. %.

**Figure 7:**
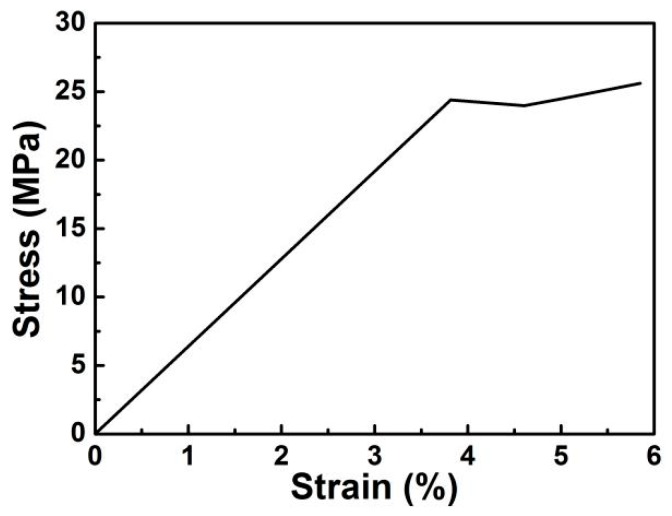
Stress-strain curve of the PAN-based gel polymer electrolyte.

One can see from Figure 7 that ultimate failure of the PAN-based gel polymer electrolyte does not occur even when it is extremely stretched up to 6%. In the same time, the tensile modulus of the mesh network is only 24.5 MPa within 3.8% strain, suggesting a high elasticity of the system. It is generally accepted that both low modulus and large elongation of the electrolytes are extremely important for batteries.

**Figure 8:**
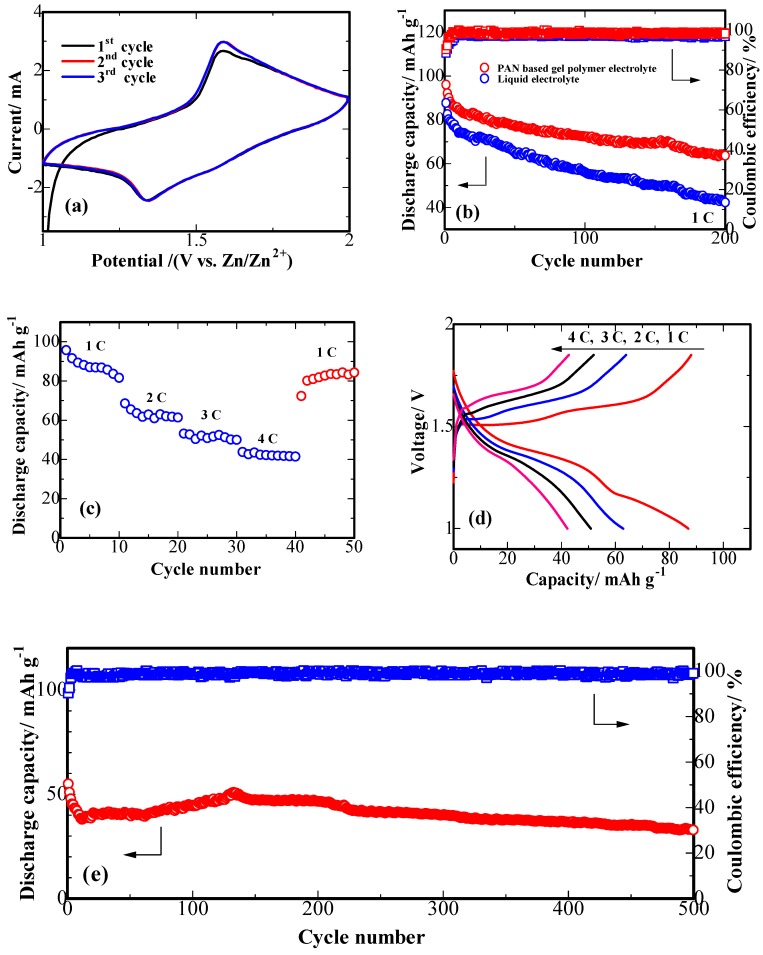
Electrochemical performance of a cell with the PAN-nanofiber-based gel polymer electrolyte of Na_4_Mn_9_O_18_ electrode (vs. Zn/Zn^2+^) (**a**) CV curves at a rate of 0.1 mV s^−1^; (**b**) Cycling performance of liquid electrolyte cell (the blue) and the PAN-nanofiber-based gel polymer electrolyte cell (the red); (**c**) Rate capability and (**d**) Charge/discharge profiles at various rates; (**e**) Prolonged cycling performance of the PAN-nanofiber-based gel polymer electrolyte cell at 4 C.

Cyclic voltammograms (CV) of Na_4_Mn_9_O_18_ and Zn electrodes are shown in Figure 8a. The upper cut-off potential was limited to 2.0 V to prevent decomposition of water [26]. The Na_4_Mn_9_O_18_ electrode shows redox peaks at ~1.35 V and ~1.59 V vs. Zn/Zn^2+^ in its discharge and charge curves, respectively, conforming to the de-insertion/insertion of Na^+^ ions in the aqueous electrolyte. The Na_4_Mn_9_O_18_ electrode possesses an excellent electrochemical reversibility which is reflected by its capability to withstand the electric current and the redox peak potentials in the same position upon the consequent cycles.

Figure 8b presents the cyclability data for the cell, which shows a low initial discharge capacity of 96 mAh g^−1^, and a low initial coulombic efficiency around 90%. Such low coulombic efficiency could be due to some irreversible side reactions in the first charging process. A cell with the PAN-nanofiber-based gel polymer electrolyte shows an excellent cycling performance (red colored curves). Figure 8c presents the data on an excellent rate performance of a cell with the PAN-nanofiber-based gel polymer electrolyte. At a low rate of 1 C, the electrode provides a reversible discharge capacity of ~88 mAh g^−1^. At the higher cycling rates of 2 C, 3 C and 4 C, the discharge capacities of ~65, 52 and 44 mAh g^−1^ were sustained, respectively. Furthermore, the electrode recovers most of its initial capacity of 84 mAh g^−1^ when the cycling rate was modulated back to 1 C, demonstrating an excellent high current abuse tolerance of our Na_4_Mn_9_O_18_ cathode. To further demonstrate the rate capability of the system, the charge/discharge profiles at various C rates are shown in Figure 8d. An increase in current densities from 1 C to 4 C cause a decrease of discharge capacity from 88 mAh g^−1^ to 44 mAh g^−1^. The electrode exhibits a good cycle stability, maintaining a specific capacity of 33 mAh g^−^^1^ after 500 cycles at 4 C as shown in Figure 8e. The electrode maintains 60% of its initial capacity after 500 cycles.

Table 1 presents the comparative performance data from the literature on the relevant/similar materials, and our PAN-nanofiber-based gel polymer electrolyte for novel aqueous sodium-ion battery exhibits a superior electrochemical performance compared with other reported systems [27,28,29].

## 4. Conclusions

In this work, Na_4_Mn_9_O_18_ nanorods were successfully synthesized by the HSCR method. A rechargeable hybrid aqueous battery employing metallic Zn as a negative electrode and Na_4_Mn_9_O_18_ as the positive electrode has been developed. The as-prepared Na_4_Mn_9_O_18_ electrode exhibited a discharge capacity of 64 mAh g^−1^ at 1 C even after 200 full cycles. The excellent cycle stability and rate performance of our Na_4_Mn_9_O_18_ material makes it a strong candidate for cathodes for rechargeable hybrid aqueous battery systems.

## Figures and Tables

**Table 1:** Literature data comparison on the electrochemical performances of gel polymer electrolytes for sodium-ion batteries.

Material	Cycle Number	Capacity Remaining (mAh g^−1^)	Current Density	Reference
PMMA-based gel polymer electrolyte	100th	68	1 C	[27]
Phosphonate-based gel polymer electrolyte	35th	117.8	1 C	[28]
Glass-fiber-based gel polymer electrolyte	100th	70	1 C	[29]
PAN-based-based gel polymer electrolyte	100th	74	1 C	This study
